# Body Mass Index, Perceived Weight, and Self-Rated Health among South Korean Adults: Conjoint Effect on Health?

**DOI:** 10.3934/publichealth.2017.5.513

**Published:** 2017-11-20

**Authors:** Soyoung Kwon

**Affiliations:** Department of Psychology & Sociology, Texas A & M University- Kingsville, 700 University Blvd, MSC 177, Kingsville, TX 78363, USA

**Keywords:** body mass index, perceived weight, intersectionality, self-rated health, Korean adults

## Abstract

The prevalence of overweight and obesity is rising rapidly in many countries, including South Korea. The present study examined the association between weight perception and self-rated health, independent of body weight status, as well as how weight status and perceived weight status intersect and relate to the self-rated health among adults in South Korea. Data were from 722 men and 800 women in 2010 Korean General Social Survey. Results showed that over half of Korean adults perceived their weight incorrectly with a fair agreement (men ƙ = 0.36; women ƙ = 0.31). Multivariate analyses indicated that poor/fair self-rated health had no significant association with body weight status, measured by self-reported weight and height, but it had a significant association with perceived weight status in men. The intersectionality analysis in which weight status and weight perceptions were cross-classified indicated that both measured and perceived weight status should be taken into account for the indicator of self-rated health as well as for better understanding of weight-related health consequences.

## 1. Introduction

The health consequences of overweight and obesity are well-documented [1]. According to pathophysiological mechanisms, excess body weight and fat exert a detrimental effect on a host of the bodily system, which leads to hypertension, metabolic syndrome, endocrinology, among other comorbidities [1]–[3]. This adiposity hypothesis contributes to the understanding of weight-related complications.

Recent studies go beyond the medical sequelae and have included the broad dimensions of health outcomes, such as psychological health (e.g., depression), perceived functional health, or Health-Related Quality of Life [4]–[6]. This body of research illustrated that weight-related stigma and discrimination provoke a stress, which in turn leads to undesirable health consequences. If there are sociocultural norms for thinness and appearance, then it is plausible to expect that perceived weight status could affect the health of individuals. Accordingly, there is a growing literature that examines the association between weight perception and health [7]–[12]. Nevertheless, a majority of public health programs and intervention around weight and weight management primarily focuses on clinically defined weight status (e.g., body mass index (BMI hereafter)) and chronic conditions.

Although increasing attention has been paid to the perceived weight status, research into independent and joint effects of weight perception with objective weight on health-related quality life, such as self-rated health is sparse. Even fewer studies examine health consequences of weight and weight perceptions among adults in non-western societies. For the society where there is a relatively low prevalence of overweight and obesity but marked discrepancies between weight and weight perceptions, self-rated health (SRH) is instructive to consider [13]. Because self-rated health subjective constructs, it would be influenced by weight perceptions, independently of and perhaps over and above objective weight. Body weight status, calculated as BMI, has been reported to be associated with self-rated health, and a robust predictor of mortality and objective physical health [14],[15].

## 2. Research Context: South Korea

It has been reported that 33% of Korean adults were overweight/obese in 2004, which is lower than 71 % of American adults who are overweight/obese rate as well as lower than the average obesity rate of 56.7% in Organization of Economic Cooperation and Development countries (OECD) [16]. However, a body weight has become a major public health concern in Korea as the number of overweight Koreans has increased 60 percent in the past decade [17]. Despite the low rate of overweight/obesity in Korea, the prevalence of comorbidities of excess weight among Korean adults is surprisingly similar to that of Americans [18]. One possible explanation is due to the lower BMI cutoff in Asian population that causes a health risk [17]. However, the present study suggests that perceived weight status may be a key to understanding this puzzle as body weight is a prevalent concern among Korean. There is also considerable disjunction between medical standards of normal body size and a cultural standard of attractive and ideal body size [13]. Therefore, a study on the role of perceived weight status is an important point of focus for the design and implementation of public health initiatives.

## 3. The Current Study

The present study proposes that the health consequences of body weight should be investigated from sociocultural perspectives because weight-related concerns (e.g., body image, body shape) and fat prejudice have been prevalent in a body conscious society. Nevertheless, little is known about the effect of perceived weight status on self-rated health. To fill the gap in the literature, this study takes an intersectional approach and investigates how weight perceptions are associated with self-rated health independently of and in combination with body mass index using samples of Korean adults from 2010 Korean General Social Survey.

This study examined how perceived weight status exerts an independent and conjoint effect on the rating of health among adults in South Korea. Specifically, the first aim of this study is to describe weight (in) congruence in a nationally representative sample of Korean adults. The second objective of this study is to examine how perceived weight status was related to poor/fair self-rated health, independent of body weight status and all other covariates. Finally, the third aim of this study is to test how body weight and perceived weight status intersects and relates to the poor/fair self-rated health. To answer the third question, nine main groups are formulated by cross-classifying participants according to BMI categories and perceived weight status.

## 4. Materials and Methods

### 4.1. Sample

This study analyzed data from 2010 Korean General Social Survey (KGSS is used hereafter), a cross-sectional study conducted in 2010 using face-to-face interviews. The KGSS used a module from a General Social Survey-type questionnaire and sampled based on a multistage stratified random sampling method, producing a nationally representative sample [19]. Response rates were 63.0%. After dropping missing cases on variables (1.03% missing of the total samples), leaving final sample size of 800 women and 722 men whose ages range from 18 to 90 and 18 to 92 years old.

### 4.2. Measures

**Body weight status.** Self-reported weight and height were used to calculate BMI with a formula, weight (kg)/height (m) ^2^. Based on World Health Organization standard BMI cut-off point, people with BMI of less than 18.5 kg/m^2^, BMI from 18.5 to less than 25 kg/m^2^ and those with a BMI of 25 kg/m^2^ or greater was considered as underweight, normal weight, and overweight/obese, respectively [20].

**Perceived weight status.** Participants were asked to report their view on body weight on five statues: (1) a lot underweight; (2) a little underweight; (3) neither underweight nor overweight; (4) a little overweight and (5) a lot overweight. These responses recoded into “perceived underweight”, “about right”, and “perceived overweight” groups in the present study [8]–[10].

**Self-rated health.** The dependent variable was an individual's self-rated health, a widely used measure of health status in the literature as it has been reported to a strong predictor of mortality and a validated and reliable indicator of general subjective health [15]. Participants were asked “How would you rate your health?” with a 5-point scale. Then, they were dichotomized into poor/fair versus excellent, very good/good health.

**Covariates.** Covariates were participant's socio-demographic characteristics, such as age, marital status (married = 1, otherwise = 0), employment status (full-time employee = 1, otherwise = 0), quintile of household income , educational attainment (< high school, high school graduates, some college, ≥ college), place of residence (city, small city, rural), and number of kids. Health and health behaviors were also included: the presence of a chronic condition (at least having one chronic disease = 1 versus no chronic disease = 0), the frequency of physical activity (5-point scale), the frequency of alcohol consumptions (5-point scale), and smoking status (current smoker = 1) (see Table 1).

**Table 1.** Sample characteristics, South Korea General Social Survey, 2010.

*Characteristics*	*Men (N=722)*		*Women (N=800)*	
	*Mean/%*	*SD*	*Mean/%*	*SD*
Poor/fair self-rated health	19%		27%	
BMI (kg/m)				
Underweight	3%		11%	
Normal weight	68%		72%	
Overweight/obesity	29%		17%	
Perceived weight status				
Underweight perception	23%		13%	
About right perception	49%		45%	
Overweight perception	28%		42%	
Age	44.71	16.2	45.09	16.45
Marital status				
Married	62%		65%	
Number of children	1.42	1.26	1.72	1.23
Residence				
City	58%		55%	
Small city	30%		31%	
Rural	12%		14%	
Education				
< high school	18%		27%	
high school	29%		32%	
Some college	16%		15%	
College graduates	38%		27%	
Employment status				
Full-time employment	62%		38%	
Family income (quintile)	20%		20%	
< 1,500,000 won				
1,500,000 won–2,990,000 won				
3,000,000 won–4,490,000 won				
4,500,000 won–7,490,000 won				
>7,490,000 won				
Physical activity				
Never	20%		47%	
Several times a year or less often	7%		30%	
Several times a month	18%		38%	
Several times a week	34%		44%	
Daily	21%		34%	
Alcohol drink				
Never	20%		43%	
Several times a year or less often	9%		19%	
Several times a month	23%		24%	
Several times a week	38%		13%	
Daily	10%		1%	
Currently smoking	50%		5%	
Presence of chronic disease	27%		33%	

### 4.3. Analysis

In the first step analyses, respondents were cross-classified by weight status (i.e., underweight, normal weight and overweight/obese) according to body mass index and perceived weight status (i.e., perceived underweight, about right, perceived overweight) to see how perceived weight status varies across BMI. Also, overall agreement between measured weight status and perceived weight status was calculated and assessed using *kappa* statistic.

Then, multivariate logistic regression analyses were performed to estimate how perceived weight status is associated with poor/fair self-rated health, independently of weight status. Specifically, the first model included only perceived weight status controlling for all covariates. Then, body weight status measured by BMI was added to a subsequent model. Another set of multivariate regression analyses focused on the joint effect of BMI weight status and perceived weight status with the following nine mutually exclusive classifications: underweight-perceived underweight perception, underweight-about right perception, normal weight-perceived underweight, normal weight-about right perception (reference group), normal weight-perceived overweight, overweight-about right perception, and overweight-perceived overweight. Estimated effects of combined body weight-perceived weight status were reported in odds ratios and predicted probabilities. Adjusted odds ratios with confidence interval were displayed in foreplot. Based on the full model with combined BMI weight status and perceived weight status, predicted probabilities of each category were generated and reported in Figure 2 to illustrate how weight and weight perceptions jointly were related to the poor/fair self-rated health. All analyses were stratified by sex [7]–[12] using Stata 14.0 [21].

## 5. Results

### 5.1. Agreement between BMI weight status and perceived weight status

Table 2 displays a cross-tabulation of weight status classified according to BMI (i.e., underweight, normal weight, and overweight/obese) and perceived weight status (e.g., perceived underweight, about right, and perceived overweight). More than half of women and men in each weight status congruently perceived their weight, however, congruent weight perception (e.g., underweight – perceived underweight) was more prevalent among underweight and overweight adults than normal weight adults.

**Table 2.** Agreement between self-reported weight status and weight perceptions among South Korean adults.

*Weight perceptions*	*BMI Classifications*			*ƙ (95% CI)*	*p value*
	*Underweight*	*Normal weight*	*Overweight*		
	*Prop. (95%CI)*	*Prop. (95%CI)*	*Prop. (95%CI)*
**Panel A. Women (N = 800)**					
Underweight	0.62 (0.52–0.73)	0.07 (0.06–0.10)	0.02 (0.004–0.063)	0.312 (0.297–0.357)	< .001
About right	0.37 (0.27–0.48)	0.53 (0.49–0.57)	0.15 (0.09–0.21)		
Overweight		0.39 (0.35–0.43)	0.83 (0.76–0.89)		
**Panel B. Men (N = 722)**					
Underweight	0.95 (0.75–0.99)	0.29 (0.25–0.33)	0.004 (0.00–0.03)	0.365 (0.331–0.395)	< .001
About right	0.05 (.001–0.25)	0.59 (0.55–0.63)	0.29 (0.23–0.36)		
Overweight		0.12 (0.09–0.15)	0.70 (0.64–0.76)		

Note: Prop.: proportion; CI: confidence interval; ƙ: kappa statistics;

Weight status: overweight (body mass index [BMI] ≥ 25.0); normal (BMI = 18.5–24.9) ; underweight (BMI < 18.5).

Gendered patterns in weight (in) congruence was also observed. Overweight women were more likely to have congruent weight perceptions than men (0.83 vs. 0.70), but underweight men were more likely to have congruent weight perceptions than women (0.95 vs. 0.62). As for the weight incongruence, 37% of women and 5% of men perceive themselves as normal weight despite being in underweight. Further, 39% of women and 12% of men perceive themselves as overweight despite being in normal weight. Taken together, men tended to underestimate their weight (i.e., perceived themselves as underweight when in fact they were classified in normal weight), while women overestimated their weight. Overall agreement between weight and weight perceptions was slightly higher for men than (ƙ 0.36) than for women (ƙ 0.31), indicating a fair agreement (e.g., < 0.21 for poor agreement; 0.21–0.40 for fair agreement; 0.41–0.60 for moderate agreement; > 0.60 for strong agreement) [22].

### 5.2. Associations between perceived weight status and self-rated health

Table 3 reports how perceived weight status was associated with poor/fair self-rated health independent of BMI weight status.

**Table 3.** Odds Ratio from Logistic Regression Models Predicting poor/fair self-rated health, KGSS 2010.

*VARIABLES*	*Women (n = 800)*		*Men (N = 722)*	
	*Model 1*	*Model 2*	*Model 1*	*Model 2*
	*Odds ratio*	*Odds ratio*	*Odds ratio*	*Odds ratio*
	*(95% CI)*	*(95% CI)*	*(95% CI)*	*(95% CI)*
**Perceived weight status**	**(ref. = about right)**			
Underweight	1.986+	2.321+	2.795***	2.500**
	(0.998–3.950)	(0.983–5.479)	(1.587–4.923)	(1.353–4.618)
Overweight	1.578*	1.323	2.671***	2.764**
	(0.999–2.494)	(0.801–2.186)	(1.546–4.616)	(1.500–5.093)
**BMI Weight status ^a^**	**(ref. = normal weight)**			
Underweight		1.431		0.404+
		(0.539–3.798)		(0.139–1.169)
Overweight/obese		2.213		0.390
		(0.701–6.985)		(0.114–1.334)
Pseudo R2	0.3585	0.3615	0.2540	0.2594
BIC	−4565.182	−4554.579	−4054.062	−4032.386

Note: CI: confidence interval; All models include control variables representing respondents' sociodemographic characteristics (age, family income, employment status, education, marital status) and health behaviors (smoking, alcohol drinking, frequency of exercise, and presence of chronic disease); ^a^ weight status was based on body mass index with self-reported height and weight; *** p < 0.001, ** p < 0.01, * p < 0.05, + p < 0.1.

Model 1 in the first column of Table 3 presents baseline relationship between perceived weight status and poor/fair self-rated health in the sample of Korean women, controlling for sociodemographic characteristics and health behaviors. The odds of poor/fair self-rated health were higher for both perceived underweight and perceived overweight in women, compared to those who perceive themselves as “about right”. The inclusion of weight status in Model 2 attenuated ORs for the perceived overweight that became statistically nonsignificant, whereas ORs for the perceived underweight remained marginally significant. For men, the odds of poor/fair SRH were significantly higher for both perceived underweight and perceived, even controlling for body mass index (see the last two columns of Table 3). In short, perceived weight status appeared to be independently associated with a rating of health in men but not in women, whereas BMI categories were not significantly related to poor/fair self-rate health.

### 5.3. Conjoint effect of body weight and perceived weight status

**Fig. 1.**
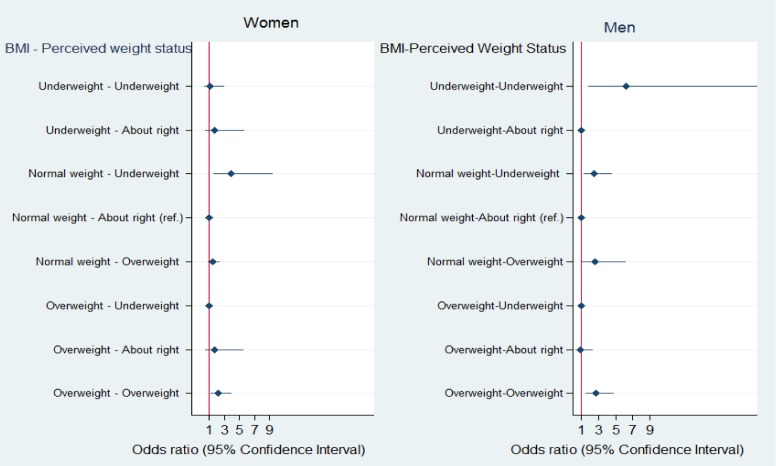
Adjusted odds (95% CI) of poor/fair self-rated health in Korean adults by combined categories of weight and weight perceptions, KGSS 2010. (Note: Odds ratios were from the regression model where nine combined weight status and perceived weight status were included as an independent variable and “normal weight-about right weight perception” as a reference group. Adjusted for sociodemographic characteristics and health-related covariates noted above.)

In the second set of regression analysis, the combined BMI-perceived weight categories were included as an independent variable to examine how body weight and perceived weight status intersects and predicts rating of health net of all other covariates. This set of analyses revealed what can be missed when models do not consider the conjoint effect of weight and weight perceptions. Results are presented in Figure 1 with adjusted ORs and confidence interval for each combined BMI-weight perceptions category. For those perceiving themselves as “about right”, an underweight or overweight made no significant difference (95% CIs overlap 1) to the odds of poor/fair SRH, compared with the ‘normal weight-about right’ reference. For normal-weight women, underweight perception increased the odds of poor/fair self-rated health. Another significant association among women was observed in the categories of overweight and overweight perception. Compared to the normal weight women who perceived themselves as about right, overweight women who perceived themselves as overweight had higher odds of poor/fair self-rated health. Turning to the men, congruence around overweight and underweight were significantly associated with higher odds of poor/fair SRH than congruence around normal weight. Also, normal weight men with perceived underweight had higher odds of poor/fair self-rated health than normal weight with “about right” weight perception.

**Fig. 2.**
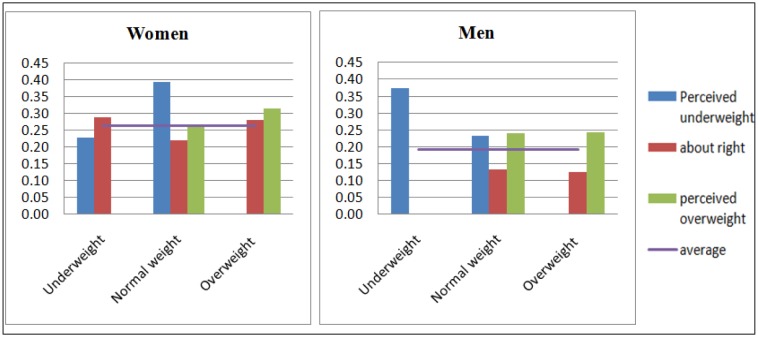
The predicted probability of reporting fair/poor SRH by combined categories of weight and weight perceptions among Korean adults. (Note: The predicted probability was generated from the logistic model with the combined Weight status – Weight perceptions and normal weight – about right weight perception as the reference group. Columns for underweight-perceived overweight women, underweight-about right weight perception men, and underweight-overweight perception men due to the small number of cases.)

To put the magnitudes of results in contexts, predicted probabilities were also generated, varying key contrasts (e.g., weight status and perceived weight) and holding other variables at their mean. The predicted probability aids us in identifying which women and men are at greatest risk of poor/fair self-rated health than average adults (indicated by the horizontal line in Figure 2). Figure 2 suggests that predicted probability of poor/fair SRH for normal weight women depends on their perceived weight status. For example, normal-weight women with perceived underweight had the highest probabilities of poor/fair self-rated health, followed by overweight-overweight perception. As expected, normal weight women with “about right” weight perception were shown to be least likely to report poor/fair health. Turning to which men were more likely to have poor/fair self-rated health than normal-weight men who perceived their weights as “about right” (the group expected to be least likely to report poor/fair SRH). It was normal weight men with overweight and underweight perception. Also, overweight men with overweight perception had an almost equally high risk of poor/fair SRH. Regardless of BMI, men with “about right” weight perception showed the lower risk of poor/fair SRH than average and other groups.

## 6. Discussion

This study examined how perceived weight status exerts an independent and conjoint effect on the rating of health among adults in South Korea. Specifically, the first aim of this study was to describe weight (in) congruence in a nationally representative sample of Korean adults. The second objective of this study was to examine how perceived weight status was related to poor/fair self-rated health, independent of body weight status and all other covariates. Finally, the third aim of this study was to test how body weight and perceived weight status intersects and relates to the poor/fair self-rated health. To answer the third question, nine main groups were formulated by cross-classifying participants according to BMI categories and perceived weight status.

On a descriptive note, adults' weight perceptions often did not agree with their actual weight status, even when they were self-reported. Overall agreement between weight perceptions and self-reported BMI was only fair (ƙ = 0.31 for women; ƙ = 0.36 for men) in Korean adult. This agreement was lower than agreement in American adults (ƙ = 0.48 for women and 0.45 for men) as well as Canadian adults (ƙ = 0.58 for women and 0.42 for men) [10], [23]. Further, nearly 40% of the Korean adults misperceived their body weight. Study with a sample of Canadian adults reported that almost 20% of adults misclassified themselves. Another recent study using a sample from Iranian children and adolescent found nearly 40% of participants misperceived their weight, suggesting that possibly adults has a closer to reality body image compared to children/adolescents [9]. However, the finding from Korean adults in the present study does not seemingly support this explanation. Rather than age, perceived weight status could be relevant to sociocultural environments. Given relatively high discrepancies between measured weight status and perceived weight status, Korea provides an opportune context for exploring health consequences of perceived weight status independently of as well as jointly with actual body weight.

In our body conscious society, body weight has been increasingly central to one's well-being and quality of life. Accordingly, increasing attention has been given to the weight perceptions and its impact on psychological well-being. The present study extends the literature to examine self-rated health as an outcome in non-western countries. The findings revealed that weight perceptions served as a significant independent predictor of self-rated health, whereas there is no significant effect of objective weight status (i.e., BMI). It may suggest that Korean adults regard weight-related body image and physical appearance as an important health assessment index [13]. A previous study showed that weight-based stigma shapes one's weight perceptions, which in turn mediate the relationship between weight discrimination and self-rated health [5]. Conceivable mediators of the link between weight perception and SRH may include self-esteem, negative body image, and psychological distress, which warrant further study for understanding its specific mechanism. Interestingly, this study failed to find a significant effect of actual weight status, measured by BMI. Presumably, low prevalence of overweight and obesity in South Korea may explain its null association.

Moving beyond a traditional approach where one or the other but both matters, the present study applied the intersectionality approach to understanding how BMI weight status and perceived weight status jointly predict and identify who is likely to report oneself to have poor or fair health. By cross-classifying respondents according to BMI categories and perceived weight status, this study illuminated the complexity that arises from adults simultaneously grappling with both a number on a scale and an image in the mirror better predicts rating of health [11].

Frisco and her colleagues [11] proposed weight congruency and double jeopardy as the competing theoretical explanations for how body weight and perceived weight status is jointly related to the depressive symptoms among adolescents in the U.S. They found that weight pessimists (perceive weight is worse than actual weight) were at greater risk for depressive symptoms, supporting weight congruency. However, the empirical findings from the present study on self-rated health among Korean adults revealed that overweight adults with overweight perceptions were at greater risk of poor/fair self-rated health. There was also a significant difference between overweight men and women with overweight perception and normal weight adults with “about right” weight perception — the most important comparison for double jeopardy model. Further, underweight men with underweight perception were at the greatest risk of poor/fair self-rated health. Theoretically, this study lends support to the double jeopardy that applies to understanding how actual weight and perceived weight status is jointly related to self-rated health among adults. Future research needs to identify a theoretical lens for the process by which how stress, perceptions, and actual weight intersect to shape various aspects of well-being over the life course.

## 7. Limitations

There are several limitations to this study. The data analyzed are cross-sectional, which limit the inference of causal relationship. In other words, we cannot rule out the reverse relationship that poor self-rated health may be a predictor of weight gain [23],[24]. In a similar vein, a person with poor self-rated health may have a negative perception about their weight. Another limitation includes the measure of key variable-body weight. As mentioned before, body mass index was measured with self-reported weight and height, which may lead to overestimating the effect of perceived weight status [25],[26]. However, it is remarkable that, even when body weight and height used for BMI was self-reported, notable discrepancies between weight and weight perceptions exist. More prospective studies are needed to examine the association between clinical or both diagnosed physical and psychological conditions, actual weight and perceived weight status.

## 8. Conclusions

The present study demonstrated that objective and perceived measures of weight status should not be treated as independent, separate construct for understanding subjective, physical health especially in a country where there are relatively high discrepancies between body mass index and perceived weight status. The findings suggest that body mass index and perceived weight status should be taken into consideration for clinician and public health policymaker interested in weight-related health promotion.
